# Association between Aqueous Cytokines and Diabetic Retinopathy Stage

**DOI:** 10.1155/2017/9402198

**Published:** 2017-06-07

**Authors:** Hailiang Wu, De-Kuang Hwang, Xudong Song, Yong Tao

**Affiliations:** ^1^Beijing Tongren Eye Center, Beijing Tongren Hospital, Capital Medical University, Beijing, China; ^2^Beijing Ophthalmology & Visual Sciences Key Laboratory, Beijing, China; ^3^Department of Ophthalmology, Taipei Veterans General Hospital, Taipei, Taiwan; ^4^Department of Ophthalmology, School of Medicine, National Yang-Ming University, Taipei, Taiwan; ^5^Department of Ophthalmology, Beijing Chaoyang Hospital, Capital Medical University, Beijing, China

## Abstract

**Purpose:**

To measure the concentrations of various cytokines in the aqueous humor from patients with different stages of diabetic retinopathy.

**Methods:**

All selected cataract patients were categorized into 4 groups: the control group (patients without diabetes), nonretinopathy (NDR) group (diabetic patients without retinopathy), nonproliferative diabetic retinopathy (NPDR) group, and proliferative diabetic retinopathy (PDR) group. The aqueous concentrations of interleukin- (IL-) 1*β*, IL-2, IL-4, IL-5, IL-6, IL-10, interferon-*γ*, tumor necrosis factor-*α*, and vascular endothelial growth factor (VEGF) from patients were measured using the cytometric bead array technique.

**Results:**

In this study, 10, 22, 15, and 14 patients were included in the control, NDR, NPDR, and PDR groups, respectively. No difference was observed in the aqueous concentrations of all cytokines between the control group and the NDR group. By contrast, comparison of these groups revealed that the aqueous concentrations of most inflammatory cytokines were significantly higher in the PDR and NPDR groups. In addition, the concentrations of IL-2, IL-5, and VEGF were higher in the PDR group than those in the NPDR group.

**Conclusions:**

Aqueous concentrations of various cytokines increased with the severity of patients' diabetic retinopathy. This finding implies that these cytokines might play a role in the progression of diabetic retinopathy.

## 1. Introduction

Diabetic retinopathy (DR), a major complication in patients with diabetes, is a leading cause of visual blindness globally [1]. Among the several pathways involved in the pathogenesis of this microvasculopathy, inflammation has been proven to play a critical role. Evidence shows that increasing concentrations of various inflammatory mediators and proinflammatory cytokines in diabetic eyes may lead to microvascular occlusion and breakdown of the blood-retinal barrier, followed by vascular leakage, capillary nonperfusion, neurodegeneration, and neovascularization [2, 3]. Research has shown that the suppression of some cytokines can protect the pathological change of retinal capillaries in animal models [4]. Therefore, measuring various cytokines in patients with different stages of DR may facilitate the evaluation of the inflammatory status at each stage and the exploration of their effects on disease progression.

The concentrations of many mediators and pathogens in intraocular tissues are different from those in systemic circulation because of the blood-eye barrier. Sampling the aqueous humor is the most effective method for evaluating the cytokines and mediators produced by intraocular tissues [5]. However, only less than 200 *μ*L of the aqueous humor can be obtained per sampling because of its total amount in the anterior chamber and surgical limitations. The aqueous concentrations of many cytokines are too low to be determined using the conventional enzyme-linked immunosorbent assay (ELISA) technique, particularly after dilution [6]. By contrast, the cytometric bead array (CBA) technique can detect such low concentrations in these samples. Through the use of this technique, multiple mediators can be quantitatively detected in a sample of 20–50 *μ*L fluid. In addition, CBA provides results with higher repeatability and sensitivity than ELISA does [7].

In the present study, we measured various inflammatory cytokines in the aqueous humor from patients with diabetes by using CBA technology in order to explore the relationship of the concentrations of these cytokines with the severity of DR.

## 2. Materials and Methods

### 2.1. Inclusion and Exclusion Criteria

In this prospective case-control study, patients who underwent cataract surgery at the Cataract Center of Beijing Tongren Hospital from March 2014 to January 2015 were divided into 4 groups and randomly selected. Patients were diagnosed with type 2 diabetes mellitus if they met the 1999 criteria of the World Health Organization, and the categorization was made before randomization on the basis of their clinical diagnoses and medical charts. For all patients, comprehensive ophthalmic examination was performed before and after cataract surgery. DR was diagnosed and classified according to the results of indirect ophthalmoscopy. Patients were excluded if they met the following criteria: (1) having other severe diabetic complications, such as nephropathy, ketoacidosis, or hyperosmotic coma; (2) having other major systemic disorders; and (3) having other ocular diseases or a history of ocular surgery including intravitreal injections or receiving laser treatment within 12 months. This study was approved by the Institutional Review Board of Beijing Tongren Hospital before it began and was conducted in adherence to the tenets of the Declaration of Helsinki. All samples were collected after patients understood and signed the informed consent for this research.

Patients were categorized into the control group if they had never been diagnosed as having diabetes or hyperglycemia. According to the preoperative (if the fundus can be well observed) or postoperative examination of fundus, the patients with diabetes were categorized into the following 3 groups: the nonretinopathy (NDR) group if they did not have any evidence of DR, nonproliferative diabetic retinopathy (NPDR) group if they had retinopathy without any retinal neovascularization, and proliferative diabetic retinopathy (PDR) group if retinal neovascularization was found in their fundus.

### 2.2. Sample Collection and Measurement

Just before cataract surgery, each studied eye received adequate anesthesia and sterilization. A 0.1 mL syringe was used to collect 100–200 *μ*L of the aqueous humor from the anterior chamber through the corneal limbus. All samples were sealed in Eppendorf tubes and stored at −80°C until CBA analysis.

All cytokines were analyzed using Becton Dickinson CBA software following the standard protocol. CBA technology was described in detail previously [7]. Human interleukin (IL)-1*β*, IL-2, IL-4, IL-5, IL-6, IL-10, interferon (IFN)-*γ*, and a tumor necrosis factor (TNF) Enhanced Sensitivity Flex Set; a human vascular endothelial growth factor (VEGF) Flex Set; and a Human Enhanced Sensitivity Master Buffer Kit (100 Tests) were used to analyze specific cytokines.

### 2.3. Statistical Analyses

The concentrations of 9 selected cytokines in each sample were recorded after CBA analysis. The patients' demographic characteristics and the cytokine concentrations among the 4 groups were compared. One-way analysis of variance, the Wilcoxon rank-sum test, and the Kruskall-Wallis test were used to compare numerical data, and the Fisher exact test was used to compare categorical data. All statistical analyses were performed using SPSS, version 19.0 for windows (SPSS Inc., Chicago, IL, USA) and the 2-sided significance level was set at .05.

## 3. Results

A total of 61 samples from 61 patients were included in this study. Among these patients, 10, 22, 15, and 14 were included in the control, NDR, NPDR, and PDR groups, respectively. No difference was observed in age, sex, and laterality among these groups (Table 1).

By contrast, significant differences were observed in the aqueous concentrations of cytokines among the 4 groups (*P* value < 0.001, Table 2).

**Figure 1:**
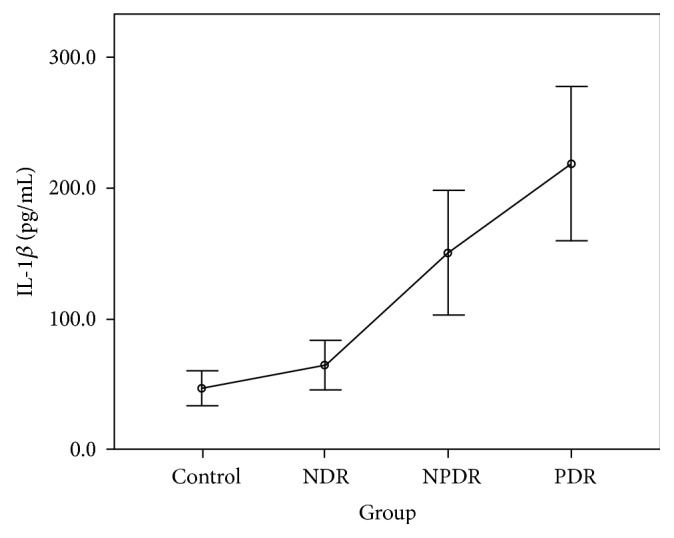
Aqueous concentration of interleukin-1*β*. Control: patients without diabetes; NDR: nondiabetic retinopathy; NPDR: nonproliferative diabetic retinopathy; PDR: proliferative diabetic retinopathy.

**Figure 2:**
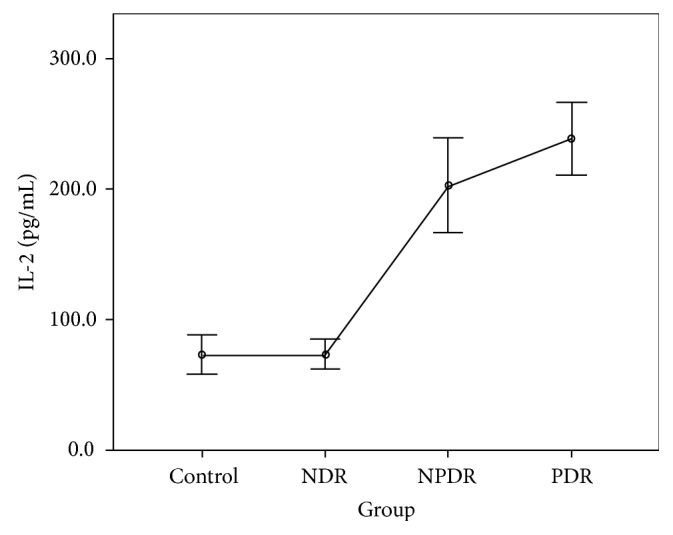
Aqueous concentration of interleukin-2. Control: patients without diabetes; NDR: nondiabetic retinopathy; NPDR: nonproliferative diabetic retinopathy; PDR: proliferative diabetic retinopathy.

**Figure 3:**
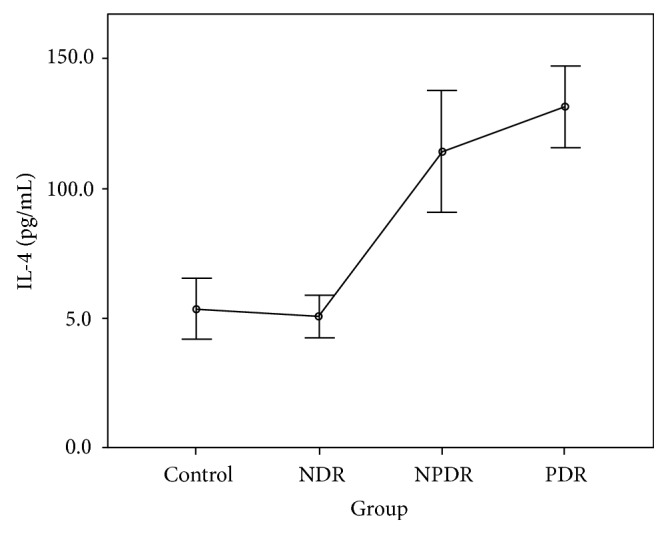
Aqueous concentration of interleukin-4. Control: patients without diabetes; NDR: nondiabetic retinopathy; NPDR: nonproliferative diabetic retinopathy; PDR: proliferative diabetic retinopathy.

**Figure 4:**
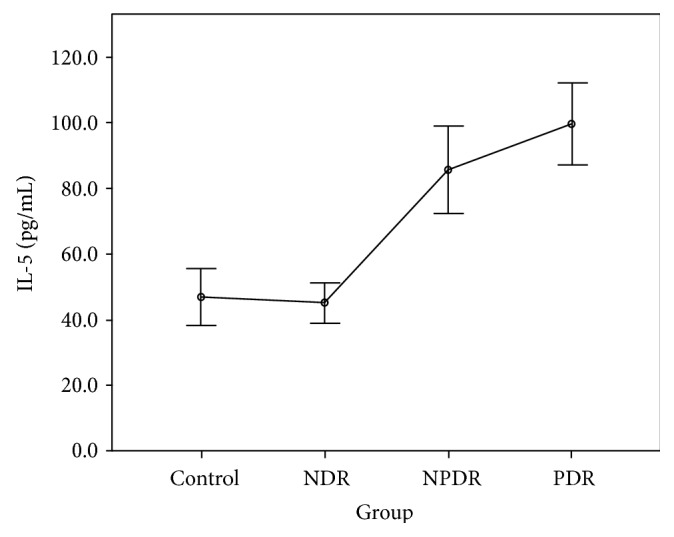
Aqueous concentration of interleukin-5. Control: patients without diabetes; NDR: nondiabetic retinopathy; NPDR: nonproliferative diabetic retinopathy; PDR: proliferative diabetic retinopathy.

**Figure 5:**
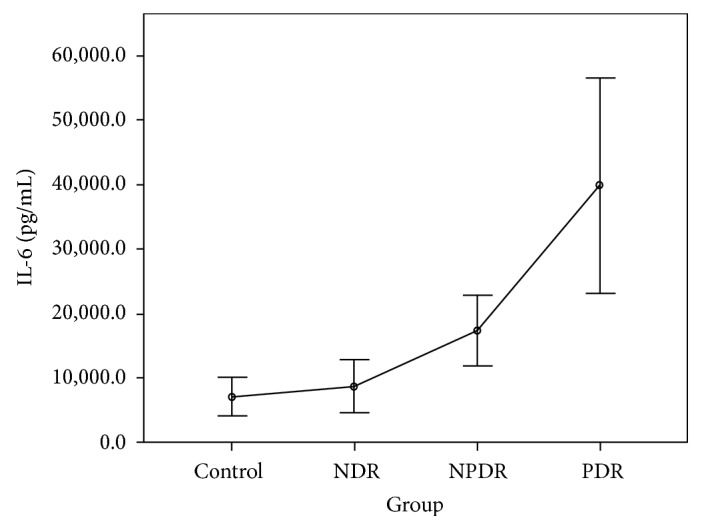
Aqueous concentration of interleukin-6. Control: patients without diabetes; NDR: nondiabetic retinopathy; NPDR: nonproliferative diabetic retinopathy; PDR: proliferative diabetic retinopathy.

**Figure 6:**
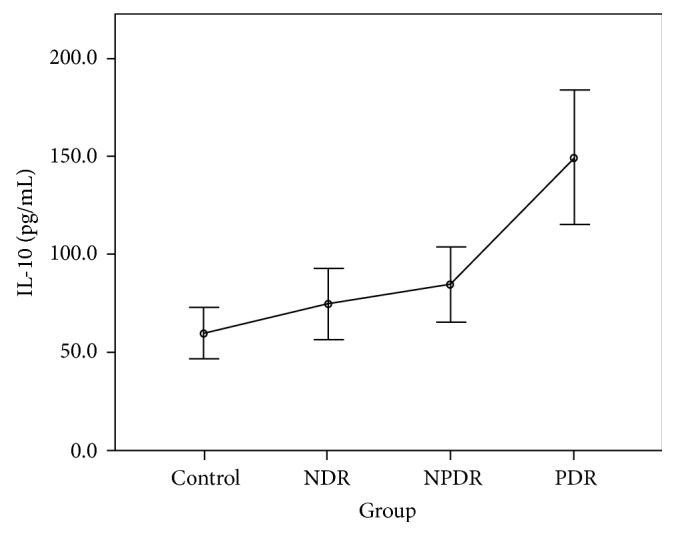
Aqueous concentration of interleukin-10. Control: patients without diabetes; NDR: nondiabetic retinopathy; NPDR: nonproliferative diabetic retinopathy; PDR: proliferative diabetic retinopathy.

**Figure 7:**
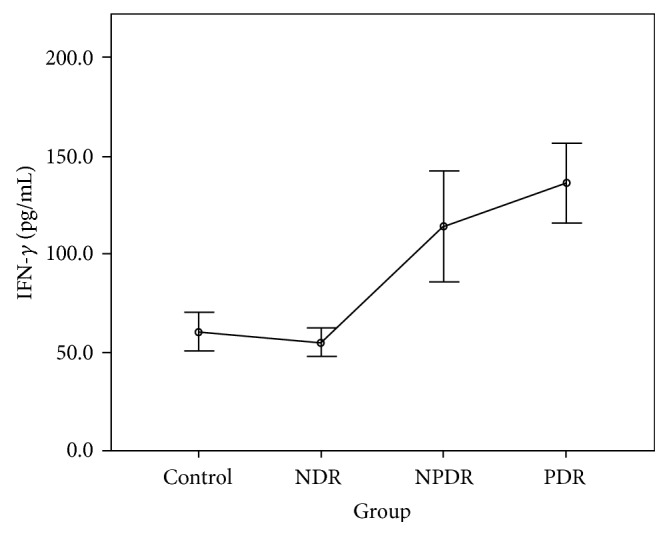
Aqueous concentration of interferon- (IFN-) *γ*. Control: patients without diabetes; NDR: nondiabetic retinopathy; NPDR: nonproliferative diabetic retinopathy; PDR: proliferative diabetic retinopathy.

**Figure 8:**
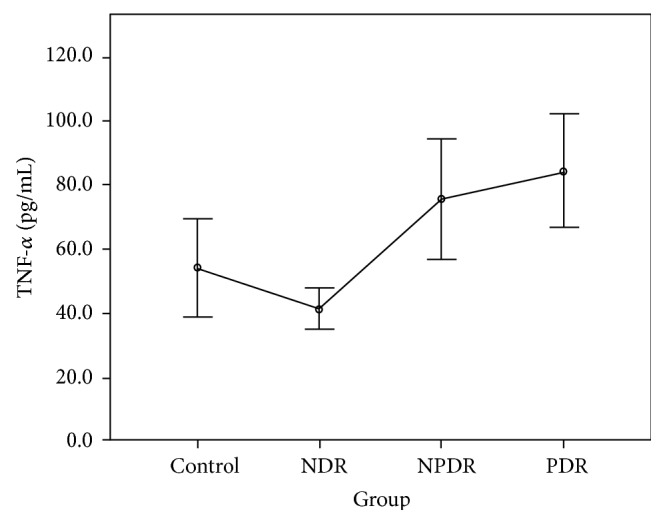
Aqueous concentration of tumor necrosis factor- (TNF-) *α*. Control: patients without diabetes; NDR: nondiabetic retinopathy; NPDR: nonproliferative diabetic retinopathy; PDR: proliferative diabetic retinopathy.

**Figure 9:**
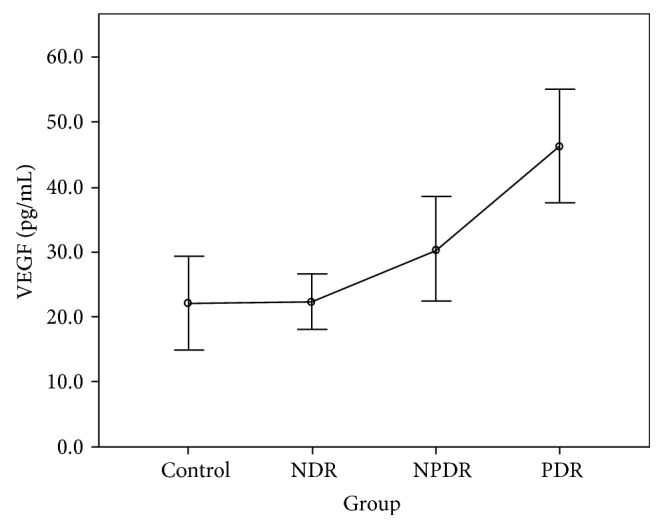
Aqueous concentration of vascular endothelial growth factor (VEGF). Control: patients without diabetes; NDR: nondiabetic retinopathy; NPDR: nonproliferative diabetic retinopathy; PDR: proliferative diabetic retinopathy.

The CBA analysis results showed no difference in the aqueous concentrations of cytokines between the control group and the NDR group. Compared with the control group, the aqueous concentrations of all cytokines were higher in those with the PDR group, and the concentrations of IL-1*β*, IL-2, IL-4, IL-5, IL-6, IFN-*γ*, and TNF-*α* were higher in those with the NPDR group. Compared with the NDR group, the aqueous concentrations of all cytokines were higher in those with the PDR group, and the concentrations of IL-1*β*, IL-2, IL-4, IL-5, IFN-*γ*, and TNF-*α* were higher in those with the NPDR group. The concentrations of IL-2, IL-5, and VEGF were higher in the PDR group than in the NPDR group (Tables 2 and 3). The aqueous concentrations of all cytokines significantly increased with the severity of DR (Figures 1–9).

## 4. Discussion

DR is a major complication in patients with diabetes. The metabolic and hemodynamic change in diabetes may lead to the nonperfusion of retinal capillaries, macular edema, neurodegeneration, and retinal neovascularization [3]. High concentrations of various inflammatory cytokines have been detected in the aqueous, vitreous, and retina samples from patients with DR. These mediators are highly related to the breakdown of the blood-retinal barrier and the development of retinal neovascularization. Hence, inflammation plays a vital role in the pathogenesis of DR [8–10]. Analyzing specific cytokines at each stage of DR may contribute to a comprehensive understanding of the inflammatory process and its correlation with the progression of DR.

Collecting and analyzing aqueous humor samples are effective and practical strategies for monitoring the intraocular concentrations of cytokines in the eye. Because of the limited volume of obtainable aqueous and the relatively high sample requirement, only few cytokines can be analyzed simultaneously by using the conventional ELISA technique. By contrast, the CBA technique used in this study can analyze up to 9 cytokines simultaneously in a volume as low as 200 *μ*L for each sample. Hence, the results of CBA analysis may provide comprehensive insights into the role of the inflammatory process in the development and progression of DR.

Among the 9 analyzed cytokines, IL-1*β*, IL-2, IFN-*γ*, and TNF-*α* are mainly produced by human CD4^+^ T-helper (Th) 1 cells, and IL-4, IL-5, IL-6, and IL-10 are mainly produced by Th2 cells. These 2 types of Th cells promote cellular as well as humoral immune responses and are highly associated with inflammation and hypersensitivity. A previous study detected cytokines produced by both Th1 and Th2 in vitreous samples [11]. In this study, we analyzed these cytokines for a comprehensive understanding of the inflammatory process in patients with DR.

Cheung et al. used a magnetic color-bead-based multiplex assay to compare various cytokines in the aqueous humor between patients with and without DR [12]. They found that the measureable rate of IL-1*β*, IL-4, and IL-5 was lower than 50%, and the aqueous concentrations of IL-2, IL-10, and TNF-*α* were lower in the patients with DR than in the non-DR control patients. In the present study, all 9 cytokines were measurable in all samples and increased with the progression of DR. The higher detection rate in this study may be attributed to the high sensitivity of the CBA technique. Our findings suggest that the degree of intraocular inflammation is positively correlated with the stage of DR.

Our results reveal no difference in the concentrations of all cytokines between the control group and the NDR group. This finding implies that the intraocular immune status might be stable, and both Th1 and Th2 cells are not activated before the development of DR. By contrast, the cytokines produced by both Th1 (IL-1*β*, IL-2, IFN-*γ*, and TNF-*α*) and Th2 (IL-4 and IL-5) cells were increased in the NPDR group. This increase might be attributed to the activation of the inflammatory process and the breakdown of the blood-eye barrier as soon as the retinopathy developed. Our finding also shows that the concentration of VEGF was not significantly elevated at this stage. Moreover, the concentrations of IL-2 and IL-5 were higher in the PDR group than in the NPDR group. This finding indicates that IL-2 and IL-5 might be associated with the development of retinal neovascularization in patients with DR [13]. The significant increasing trend in our study proved the positive correlation of the inflammatory status with the degree of retinopathy. We suggest that these inflammatory mediators not only resulted from but also aggravated the patients' DR.

IL-1*β* has been proved to be a crucial mediator associated with early retinal damage, and this cytokine has been found to be elevated in the vitreous of patients with DR. The increasing aqueous concentration of IL-1*β* found in the NPDR group in this study might promote the inflammatory process initially and trigger the production of other cytokines [14, 15]. Previous studies have detected a high concentration of IL-2 in the serum and the epiretinal membrane of patients with DR [16, 17]. Although some studies have suggested the antiangiogenetic and protective effects of IL-2, we found that the concentration of IL-2 increased with the progression of DR in our study [9, 18]. IL-4 may antagonize other proinflammatory processes, suppress neovascularization, reduce cellular damage caused by immune processes, and protect retinal ganglion cells [4, 19]. However, we found that this cytokine was significantly increased in the NPDR group, but the increase became nonsignificant when DR progressed to PDR. The concentrations of IL-6 and IL-10 increased gradually with the degree of DR in our study. The increasing trends were not as significant as those of other cytokines. We thus suggest that IL-6 and IL-10 might interact with other mediators but do not play the main role in the progression of DR.

As the first mediator proven to affect the vascular endothelium, IFN-*γ* might interact with other proinflammatory and angiogenetic factors and play a vital role in the pathogenesis of DR [12]. According to our review of the literature, no study has measured IFN-*γ* in vitreous or aqueous samples from patients with PDR [16, 20]. Using the CBA technique, we quantitatively detected IFN-*γ* in all samples and found that this cytokine increased with the degree of DR. In addition, our study revealed the positive correlation of TNF-*α* with DR, confirming its importance shown in previous studies [15, 21–23]. Finally, our study found that the concentration of VEGF increased gradually as NDR progressed to NPDR but significantly as NPDR progressed to PDR, consistent with previous research and clinical findings in DR [24].

Our study has several limitations. First, the medical and pharmacological histories of patients were self reported, and DR was classified using indirect ophthalmoscopy rather than fluorescence angiography. The degree of DR might have been underestimated in some patients. However, because the bias is toward the null, we believe that this does not alter our findings. Second, we collected only aqueous humor samples in this study. Without detailed information regarding the cytokine concentrations in systemic blood or vitreous, we did not know whether these increased cytokines resulted from intraocular inflammation or infiltration through the disrupted blood-eye barrier. In addition, plasma HbA1c was not routinely measured in this study, which may result in incomplete understanding of the status of diabetes. Finally, we could not definitely determine whether the change in cytokine concentrations was caused by the disease process or disease progression in this study.

In conclusion, we observed that various inflammatory cytokines and mediators increased with the degree of DR. Analyzing the intraocular concentrations of specific cytokines is crucial for monitoring the inflammatory status and clinical severity of DR. Research regarding the accurate measurement of more proinflammatory cytokines and their relationship with the progression of DR should be conducted.

## Figures and Tables

**Table 1:** Clinical information of cases.

Group	Cases (*n*)	Mean age (yrs)	Sex (*n*)		Diabetes duration (mean, yrs)	Preoperative blood glucose (mmol/L)	Eye (*n*)	
			Male	Female			Right	Left
Control group	10	72	4	6	0	5.74–1.26	7	3
NDR group	22	68	4	18	13	8.54–2.47	11	11
NPDR group	15	66	7	8	15	9.56–3.53	10	5
PDR group	14	67	6	8	17	8.01–1.83	7	7
*P* value		0.314^a^	0.015^b^		0.248^a#^	0.290^a#^	0.249^b^	

^a^Analysis of variance; ^b^Chi-square test; ^#^comparison among the NDR, NPDR, and PDR groups; NDR: nondiabetic retinopathy; NPDR: nonproliferative diabetic retinopathy; PDR: proliferative diabetic retinopathy.

**Table 2:** Concentrations of cytokines in aqueous humor among groups.

Groups	IL-1*β* (pg/L)	IL-2 (pg/L)	IL-4 (pg/L)	IL-5 (pg/L)	IL-6 (pg/L)	IL-10 (pg/L)	IFN-*γ* (pg/L)	TNF-*α* (pg/L)	VEGF (pg/L)
Control group	46.88 ± 18.38	72.81 ± 21.18	53.65 ± 16.13	47.02 ± 12.12	7107.10 ± 4189.20	60.07 ± 17.82	60.29 ± 14.17	54.20 ± 21.15	22.11 ± 10.11
NDR group	64.89 ± 42.52	73.34 ± 26.05	50.93 ± 18.31	45.17 ± 14.00	8740.87 ± 8892.68	74.98 ± 40.32	54.96 ± 16.29	41.29 ± 13.94	22.38 ± 9.45
NPDR group	150.63 ± 85.97	202.79 ± 65.46	114.10 ± 42.10	85.71 ± 23.89	17333.24 ± 9716.42	84.56 ± 34.17	114.26 ± 50.76	75.72 ± 33.73	30.28 ± 14.53
PDR group	218.25 ± 102.36	238.11 ± 48.23	131.24 ± 26.87	99.61 ± 21.58	39859.28 ± 28786.05	149.22 ± 59.22	136.36 ± 35.55	84.35 ± 30.82	46.31 ± 15.17
*F* value	37.16^a^	58.88^b^	34.00^b^	33.72^b^	27.58^a^	21.37^a^	23.70^b^	10.37^b^	12.22^b^
*P* value	<0.001^a^	<0.001^b^	<0.001^b^	<0.001^b^	<0.001^a^	<0.001^a^	<0.001^b^	<0.001^b^	<0.001^b^

^a^Kolmogorov-Smirnov test; ^b^analysis of variance; NDR: nondiabetic retinopathy; NPDR: nonproliferative diabetic retinopathy; PDR: proliferative diabetic retinopathy.

**Table 3:** Comparison of concentrations of cytokines between groups.

	*P* value					
	Control versus NDR	Control versus NPDR	Control versus PDR	NDR versus NPDR	NDR versus PDR	NPDR versus PDR
IL-1*β*^a^	1.000	0.001^∗∗^	<0.001^∗∗^	0.004^∗∗^	<0.001^∗∗^	1.000
IL-2^b^	0.975	<0.001^∗∗^	<0.001^∗∗^	<0.001^∗∗^	<0.001^∗∗^	0.034^∗^
IL-4^b^	0.798	<0.001^∗∗^	<0.001^∗∗^	<0.001^∗∗^	<0.001^∗∗^	0.101
IL-5^b^	0.794	<0.001^∗∗^	<0.001^∗∗^	<0.001^∗∗^	<0.001^∗∗^	0.048^∗^
IL-6^a^	1.000	0.046^∗^	<0.001^∗∗^	0.264	0.001^∗∗^	0.241
IL-10^a^	1.000	0.487	<0.001^∗∗^	1.000	0.001^∗∗^	0.051
IFN-*γ*^b^	0.668	<0.001^∗∗^	<0.001^∗∗^	<0.001^∗∗^	<0.001^∗∗^	0.072
TNF-*α*^b^	0.186	0.041^∗^	0.006^∗∗^	<0.001^∗∗^	<0.001^∗∗^	0.362
VEGF^b^	0.955	0.112	<0.001^∗∗^	0.062	<0.001^∗∗^	0.001^∗∗^

^∗∗^ *P* < 0.01; ^∗^*P* < 0.05; ^a^Kolmogorov-Smirnov test; ^b^analysis of variance; NDR: nondiabetic retinopathy; NPDR: nonproliferative diabetic retinopathy; PDR: proliferative diabetic retinopathy.
